# COVID-19-Associated Nephropathy: A Devastating Complication

**DOI:** 10.7759/cureus.43558

**Published:** 2023-08-16

**Authors:** Andrew Kim, Abdullahi Mahgoub, Abinash Parajuli, Basim Towfiq

**Affiliations:** 1 Internal Medicine, Hurley Medical Center/Michigan State University College of Human Medicine, Flint, USA

**Keywords:** nephrotic, sars-cov-2, covan, collapsing fsgs, covid-19, glomerulosclerosis, nephropathy

## Abstract

With the rapid emergence and prevalence of SARS-CoV-2 worldwide, cases of COVID-19-associated nephropathy (COVAN) from collapsing focal segmental glomerulosclerosis (cFSGS) have been reported, and the associations between the two are actively being studied. Creating appropriate treatment guidelines for COVAN requires further understanding of the pathophysiology of this type of kidney injury. This case report outlines the case of a 77-year-old patient admitted to the hospital for COVID-19 infection with a subsequent renal biopsy indicating cFSGS, adding to the data exploring the relationship between COVID-19 infections, cFSGS and the associated risk factors. Current guidelines on the treatment of COVAN are similar to those of other causes of cFSGS but continue to have poor outcomes and resistance to treatments. Further research needs to be done on both the clinical assessment and pathophysiology of COVAN to provide timely and life-saving interventions.

## Introduction

During the emergence of the COVID-19 pandemic, literature highlighted the cardiopulmonary consequences of the virus, such as acute respiratory distress syndrome and myocarditis [1]. Additional observations recognize the systemic response to COVID infection that spreads to numerous organs, including the kidney. The most common associated kidney condition reported has been acute kidney injury, with a subset that develops cFSGS, a disease well associated with other viruses, including CMV, EBV, Parvo B19, and HIV [2]. However, more research needs to be done in terms of the mechanistic similarities and differences between COVID and the other known viruses that cause cFSGS. Most patients with COVAN progress to end-stage renal disease (ESRD), and current treatments have poor outcomes and inconsistent efficacy [3]. This case report covers a patient who developed cFSGS after a COVID-19 infection.

## Case presentation

An African American woman in her seventies presented to the ED with fever, cough, malaise, and diarrhea for three days. Pertinent medical history includes chronic lymphocytic leukemia, CKD Stage IIIB, hypertension, and gout. Her vitals were pertinent for a fever of 39.6°C and a blood pressure of 178/80. The only abnormal physical examination finding was increased confusion. Ten days before her admission, the patient tested positive for COVID-19, confirmed by PCR in the ED. A chest X-ray indicated mild vascular congestion, suggesting mild congestive heart failure and pneumonia. The patient was started on broad-spectrum coverage, and once cultures and PCR indicated MRSA, only renally dosed vancomycin was continued. As seen in Table 1, the patient also experienced acute kidney injury at CKD stage IIIB.

**Table 1:** Progression of kidney function from patient’s baseline to admission to inpatient. BUN: Blood urea nitrogen, eGFR: Estimated glomerular filtration rate

	Baseline	On Admission	Reference Range
BUN (mg/dL)	40	81	7-30
Creatinine (mg/dL)	1.6	5.4	0.7-1.2
Urine Pr/Cr ratio (mg/mg)	0.36	9.5	< 0.2
eGFR (mL/min/1.73 m^2^)	34	8	> 60

**Figure 1:**
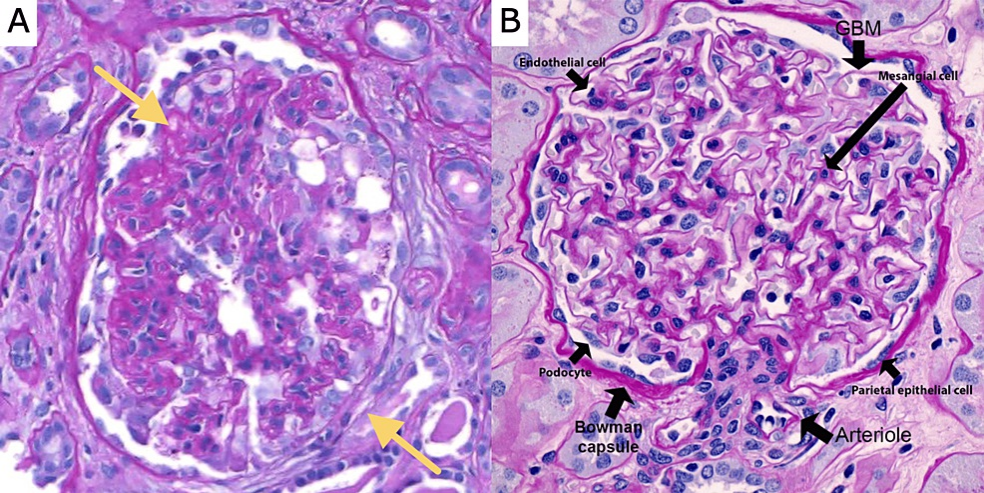
Kidney biopsy of patient (A) indicates characteristics of cFSGS, including extensive obliterated glomerular tufts (top left arrow) and collapse of the basement membrane (bottom right arrow). Normal glomerulus (B) obtained from an outside source [5].

**Figure 2:**
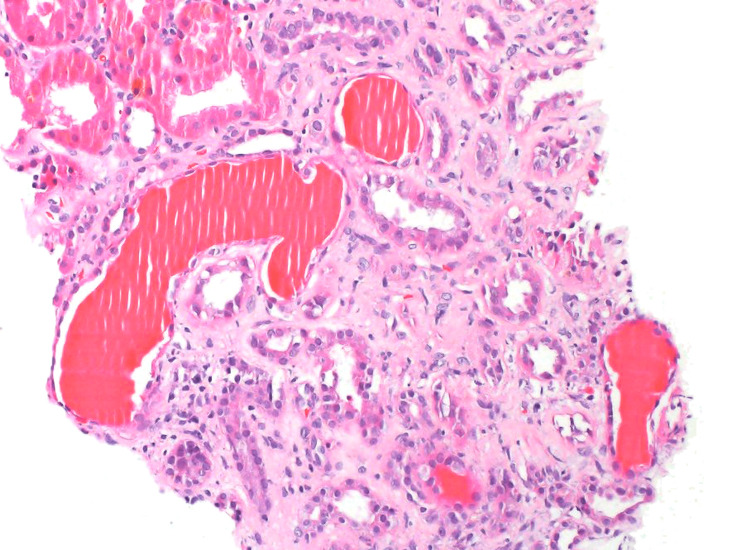
Microcystic tubular dilation is associated with virus-associated nephropathy.

This patient was compliant with medication, had well-controlled blood pressure with lisinopril and hydrochlorothiazide, and consistently saw her nephrologist. Although her pressure was high on admission, records from previous clinic appointments indicated blood pressures less than 130/80. Due to her acutely deteriorating kidney function, a biopsy was ordered, which indicated cFSGS (Figure 1). Evidence of atrophic tubules with focal microcystic dilatation (Figure 2) seen in this patient is consistent with other reported cases of COVAN [4]. Additional findings include podocyte effacement on electron microscopy and no evidence of immune deposits on immunofluorescence. Imaging, as well as the patient's history, support that her CKD is the result of hypertension and no immune process. The presence and extent of tubular atrophy and interstitial fibrosis indicate the likely progression to ESRD (Table 2).

**Table 2:** Pathology report of the patient's kidney biopsy outlining the extent of kidney injury.

Chronicity Summary	
Total Glomeruli	8
Global Glomerulosclerosis	0
Segmental Sclerosis	Present
Interstitial Fibrosis	Severe
Tubular Atrophy	Severe
Arterial Intimal Fibrosis	Severe
Arteriolar Hyalinosis	Moderate

Prednisone was started with a 12-week taper. Due to the patient's critical kidney function and lack of clinical improvement, a permcath was placed for permanent hemodialysis. The patient was discharged on oral antibiotics and scheduled for outpatient dialysis. She was readmitted to the hospital one month later, secondary to sepsis from permcath placement, and died a week later.

## Discussion

COVAN has been documented in hospitalized patients most frequently with the following risk factors: chronic kidney disease (CKD), APOL1 genotype risk common in African-Americans, and increased age [6], all of which this patient had. Current ideas suggest numerous similarities between COVAN and cFSGS caused by other viruses, which primarily act through cytokine injury to podocytes via an inflammatory reaction that upregulates interleukins and other inflammatory markers. Other proposed mechanisms explore the interaction of the renin-aldosterone system and the ACE2 protein, present in both the lungs and podocytes, as ways of viral entry into the cell [7]. This idea opens up the implications for therapeutic treatment of ACE inhibitors in acute COVID infection, but no studies to date have established a benefit in the prognosis of COVID hospitalizations.

Studies have also shown that the APOL1 genotype increases the likelihood of developing COVAN, mostly in the African American population. Podocyte expression of the APOL1 protein has been associated with kidney damage and the progression of CKD [8]. However, in this patient, APOL1 testing would not have changed the treatment plan and was not done. Further exploration of the genetic associations of COVAN allows for treatment options in both gene therapy and treatments targeted at the APOL1 protein. Currently, there are no efficacious treatments for COVAN.

The first-line treatment for cFSGS includes glucocorticoids, but many cases continue to be resistant to such treatments. As in this patient, steroids were inadequate and resulted in permanent ESRD that required a permcath placement for dialysis, which harbored an infection she ultimately died from. Current treatment guidelines for COVID nephropathy remain ineffective [9], but with further understanding of the disease, treatments can be developed to save future patients from irreversible kidney injury.

## Conclusions

The discussion of clinical presentations and risk factors for COVAN adds to the current understanding of both the cause and potential treatments of this disease. As more findings and conversations are added to the growing body of literature surrounding COVAN, attention is needed to address treatments and gain earlier recognition for this kidney pathology. Risk factors to be aware of include elderly age, African American heritage, and kidney disease. By discovering the mechanisms of injury and establishing the association, we can get one step closer to providing improved treatments for this debilitating disease. 

## References

[1] Li N, Zhu L, Sun L, Shao G (2021). The effects of novel coronavirus (SARS-CoV-2) infection on cardiovascular diseases and cardiopulmonary injuries. Stem Cell Res.

[2] Nasr SH, Kopp JB (2020). COVID-19-associated collapsing glomerulopathy: an emerging entity. Kidney Int Rep.

[3] Mubarak M (2012). Collapsing focal segmental glomerulosclerosis: current concepts. World J Nephrol.

[4] Roy S, Kunaparaju S, Koduri NM (2021). COVID-19 and APOL-1 high-risk genotype-associated collapsing glomerulonephritis. Case Rep Nephrol.

[5] Anthony Chang, MD MD (2023). Renal Fellow Network: Kidney biopsy of the month: What is normal?. https://www.renalfellow.org/2019/01/04/kidney-biopsy-of-the-month-what-is-normal/.

[6] Sabaghian T, Kharazmi AB, Ansari A (2022). COVID-19 and acute kidney injury: a systematic review. Front Med (Lausanne).

[7] Legrand M, Bell S, Forni L, Joannidis M, Koyner JL, Liu K, Cantaluppi V (2021). Pathophysiology of COVID-19-associated acute kidney injury. Nat Rev Nephrol.

[8] Reidy KJ, Hjorten R, Parekh RS (2018). Genetic risk of APOL1 and kidney disease in children and young adults of African ancestry. Curr Opin Pediatr.

[9] Giannini G, Carlos Q Velez J, May RM (2022). Renal prognosis of COVID-19 associated nephropathy. Kidney Int Rep.

